# Supplementary material for: Optimized Small Waterbird Detection Method Using Surveillance Videos Based on YOLOv7
Source: Animals (Basel). 2023 Jun 9;13(12):1929. doi: 10.3390/ani13121929 (PMC10295383; doi:10.3390/ani13121929)
Supplement: Supplementary file 1 [file animals-13-01929-s001.zip › animals-2380099-supplementary.pdf]

Supplementary Materials

Table S1. Species name and number of images in the Waterbird Dataset.

Scientific name	Common name	Number of Images
Egretta garzetta	Little Egret	169
Ardea cinerea	Grey Heron	165
Anas zonorhyncha	Chinese Spot-billed Duck	140
Nycticorax nycticorax	Black-crowned Night Heron	126
Recurvirostra avosetta	Pied Avocet	116
Anser fabalis	Taiga Bean Goose	113
Cygnus columbianus	Tundra Swan	98
Fulica atra	Common Coot	94
Larus ridibundus	Black-headed Gull	87
Mergellus albellus	Smew	85
Anser anser	Greylag Goose	78
Anas crecca	Eurasian Teal	72
Turdus merula	Common Blackbird	68
Gallinula chloropus	Common Moorhen	67
Spilopelia chinensis	Spotted Dove	66
Larus vegae	Vega Gull	65
Anas platyrhynchos	Mallard	61
Acridotheres cristatellus	Crested Myna	60
Podiceps cristatus	Great Crested Grebe	60
Phalacrocorax carbo	Great Cormorant	58
Tringa nebularia	Common Greenshank	58
Himantopus himantopus	Black-winged Stilt	57
Anas falcata	Falcated Duck	54
Streptopelia orientalis	Oriental Turtle Dove	53
Vanellus cinereus	Grey-headed Lapwing	50
Grus grus	Common Crane	48
Tachybaptus ruficollis	Little Grebe	45
Mergus merganser	Common Merganser	45
Platalea leucorodia	Eurasian Spoonbil	44
Phasianus colchicus	Common Pheasant	44
Motacilla alba	White Wagtail	44
Tadorna ferruginea	Ruddy Shelduck	43
Botaurus stellaris	Great Bittern	42
Pica pica	Common Magpie	41
Vanellus vanellus	Northern Lapwing	33
Ciconia boyciana	Oriental Stork	31
Lanius schach	Long-tailed Shrike	30
Aythya nyroca	Ferruginous Pochard	29
Gallinago gallinago	Common Snipe	27
Spodiopsar sericeus	Red-billed Starling	26
Charadrius alexandrinus	Kentish Plover	25
Passer montanus	Saxaul Sparrow	22
Anser albifrons	Greater White-fronted Goose	19
Sterna hirundo	Common Tern	18

Tadorna tadorna	Common Shelduck	17
Anas acuta	Northern Pintail	17
Tringa erythropus	Spotted Redshank	17
Aythya fuligula	Tufted Duck	16
Pycnonotus sinensis	Light-vented Bulbul	15
Anser erythropus	Lesser White-fronted Goose	15
Anas clypeata	Northern Shoveler	13
Podiceps nigricollis	Black-necked Grebe	13
Charadrius dubius	Little Ringed Plover	12
Actitis hypoleucos	Common Sandpiper	12
Glareola maldivarum	Oriental Pratincole	11
Ardeola bacchus	Chinese Pond Heron	9
Phoenicurus auroreus	Daurian Redstart	9
Tringa stagnatilis	Marsh Sandpiper	9
Limosa limosa	Black-tailed Godwit	9
Anas querquedula	Garganey	9
Aythya ferina	Common Pochard	8
Tringa ochropus	Green Sandpiper	8
Anas penelope	Eurasian Wigeon	8
Ceryle rudis	Pied Kingfisher	6
Anser cygnoides	Swan Goose	6
Amaurornis akool	Brown Crake	6
Charadrius mongolus	Lesser Sand Plover	6
Alcedo	Kingfisher	5
Emberiza chrysophrys	Yellow-browed Bunting	5
Cyanopica cyanus	Azure-winged Magpie	4
Cygnus atratus	Black Swan	3
Amaurornis phoenicurus	White-breasted Waterhen	3
Halcyon smyrnensis	White-throated Kingfisher	3
Elanus caeruleus	Black-winged Kite	3
Rallus aquaticus	Water Rail	2
Ciconia nigra	Black Stork	2
Butorides striata	Striated Heron	2
Hydrophasianus chirurgus	Pheasant-tailed Jacana	1
Pelecanus crispus	Dalmatian Pelican	1
Rallina fasciata	Red-legged Crake	1
Columba livia domestica	Domestic pigeon	1
Chlidonias leucopterus	White-winged Tern	1
Bubulcus coromandus	Eastern Cattle Egret	1
Ardea purpurea	Purple Heron	1
Tringa totanus	Common Redshank	1
Calidris alpina	Dunlin	1
Chlidonias hybrida	Whiskered Tern	1
Corvus sp.	Crow	1
Milvus migrans	Black Kite	1
Gelochelidon nilotica	Gull-billed Tern	1
Tringa glareola	Wood Sandpiper	1
Ichthyaetus ichthyaetus	Pallas's Gull	1
Sternula albifrons	Little Tern	1

Gallinago megala	Swinhoe's Snipe	1
Calidris ferruginea	Curlew Sandpiper	1
Anser indicus	Bar-headed Goose	1
Porzana pusilla	Baillon's Crake	1
Numenius phaeopus	Whimbrel	1
Aythya baeri	Baer's Pochard	1
